# SynLeGG: analysis and visualization of multiomics data for discovery of cancer ‘Achilles Heels’ and gene function relationships

**DOI:** 10.1093/nar/gkab338

**Published:** 2021-05-17

**Authors:** Mark Wappett, Adam Harris, Alexander L R Lubbock, Ian Lobb, Simon McDade, Ian M Overton

**Affiliations:** Patrick G Johnston Centre for Cancer Research, Queen's University Belfast, Belfast BT9 7AE, UK; Drug Discovery, Almac Discovery Ltd, Belfast BT9 7AE, UK; Patrick G Johnston Centre for Cancer Research, Queen's University Belfast, Belfast BT9 7AE, UK; Department of Biochemistry, Vanderbilt University, Nashville, TN 37232, USA; Drug Discovery, Almac Discovery Ltd, Belfast BT9 7AE, UK; Patrick G Johnston Centre for Cancer Research, Queen's University Belfast, Belfast BT9 7AE, UK; Patrick G Johnston Centre for Cancer Research, Queen's University Belfast, Belfast BT9 7AE, UK

## Abstract

Achilles’ heel relationships arise when the status of one gene exposes a cell's vulnerability to perturbation of a second gene, such as chemical inhibition, providing therapeutic opportunities for precision oncology. SynLeGG (www.overton-lab.uk/synlegg) identifies and visualizes mutually exclusive loss signatures in ‘omics data to enable discovery of genetic dependency relationships (GDRs) across 783 cancer cell lines and 30 tissues. While there is significant focus on genetic approaches, transcriptome data has advantages for investigation of GDRs and remains relatively underexplored. SynLeGG depends upon the MultiSEp algorithm for unsupervised assignment of cell lines into gene expression clusters, which provide the basis for analysis of CRISPR scores and mutational status in order to propose candidate GDRs. Benchmarking against SynLethDB demonstrates favourable performance for MultiSEp against competing approaches, finding significantly higher area under the Receiver Operator Characteristic curve and between 2.8-fold to 8.5-fold greater coverage. In addition to pan-cancer analysis, SynLeGG offers investigation of tissue-specific GDRs and recovers established relationships, including synthetic lethality for SMARCA2 with SMARCA4. Proteomics, Gene Ontology, protein-protein interactions and paralogue information are provided to assist interpretation and candidate drug target prioritization. SynLeGG predictions are significantly enriched in dependencies validated by a recently published CRISPR screen.

## Graphical Abstract

**Graphical Abstract:**
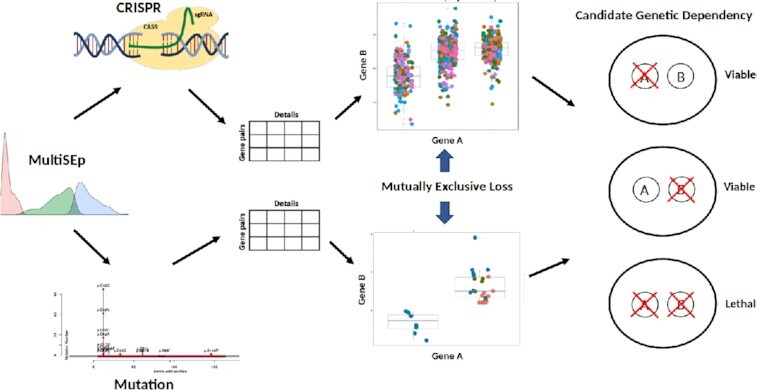
SynLeGG utilizes MultiSEp gene expression clusters to partition CRISPR essentiality scores (top) and mutations from whole exome sequencing (bottom), in order to predict candidate genetic dependency relationships, including synthetic lethality.

## INTRODUCTION

Synthetic lethality arises when loss of function (LOF) events in two or more genes results in cell death, and if cells remain viable where any one of these events occurs in isolation (1,2). Gene dependency relationships, including synthetic lethality, may produce cancer ‘Achilles heels’; indeed, cancer cells typically accumulate large numbers of genetic aberrations and therefore are vulnerable to therapeutic strategies that exploit gene dependencies (1,3,4). A striking example is where LOF mutations of homologous recombination genes BRCA1 or BRCA2, results in a dependency on DNA repair by the PARP genes and so make cells exquisitely sensitive to pharmacological inhibition of PARP1/2 (5–7). A burgeoning number of synthetic lethal relationships are well established, including within complexes such as SWI/SNF (SMARCA2/SMARCA4; ARID1A/ARID1B) (8,9); and as a collateral consequence of deletions associated with loss of tumour suppressors, for example ENO1/ENO2 (10). Other classes of genetic dependencies have been reported where gene expression plays an important role, one example is where cells ‘addicted’ to one gene (ERBB2) have decreased viability when the expression of a second gene (TFAP2C) is reduced (11). SynLeGG predicts several types of negative genetic dependencies, including synthetic lethality, in order to propose candidate Achilles’ heel vulnerabilities in cancers.

Public genome-wide RNAi and CRISPR screen data gives significant impetus to the discovery of candidate genetic dependency relationships (1,12,13). Existing web resources facilitate exploration of these screens alongside other large datasets; notably the Cancer Dependency Map (DepMap) portal, PICKLES, the Open Targets Platform and cBioPortal (1,13–16). The DepMap Data Explorer allows visualization of pairwise relationships between gene essentiality estimates and many other features including compounds, miRNA, gene expression, drug sensitivity, histone marks, metabolomics and copy number (1). A selected pairwise relationship may be analysed within the DepMap Data Explorer by linear regression or Pearson correlation; two-class comparison is also available for user-defined groups of cell lines, for example allowing exploration of tissue-specific gene essentiality. PICKLES provides visualization of gene essentiality profiles by tissue, including pairwise comparisons with orthogonal data, such as essentiality with gene expression; Pearson correlation is reported for a Bayesian gene essentiality estimate with data selected for the second gene, for example expression values (14). Additionally, PICKLES examines tissue-specific effects with a Mann-Whitney test of the difference in essentiality estimate values for individual tissue types against values calculated across all tissues. The Open Targets Platform provides a functional summary of queried genes, underpinned by multiomics data, with druggability scoring information to help prioritize targets and candidate synthetic lethal relationships (15). Overall, current web resources offer relatively simple metrics to explore potential gene dependencies. More sophisticated integrative approaches for comprehensive prediction of dependency relationships in multiomics data include DAISY, BiSEp and collective matrix factorization techniques (gCMF) (10,17,18); interaction with these approaches currently requires skills in computer programming and data handling, alongside appropriate computing resources. We developed SynLeGG (Synthetic Lethality using Gene expression and Genomics; www.overton-lab.uk/synlegg) for discovery and visualization of cancer ‘Achilles heel’ relationships with integrated, matched RNA-Seq, CRISPR, exome sequencing and mass spectrometry proteomics data (1,13,19,20). Transcriptome data is very informative for identification of pairwise gene dependencies (2,12) and is taken as a central axis in SynLeGG, across 783 cell lines and 30 tissues.

## METHODS

### Predicting gene dependency relationships from CRISPR and mutational data with MultiSEp

**Figure 1.**
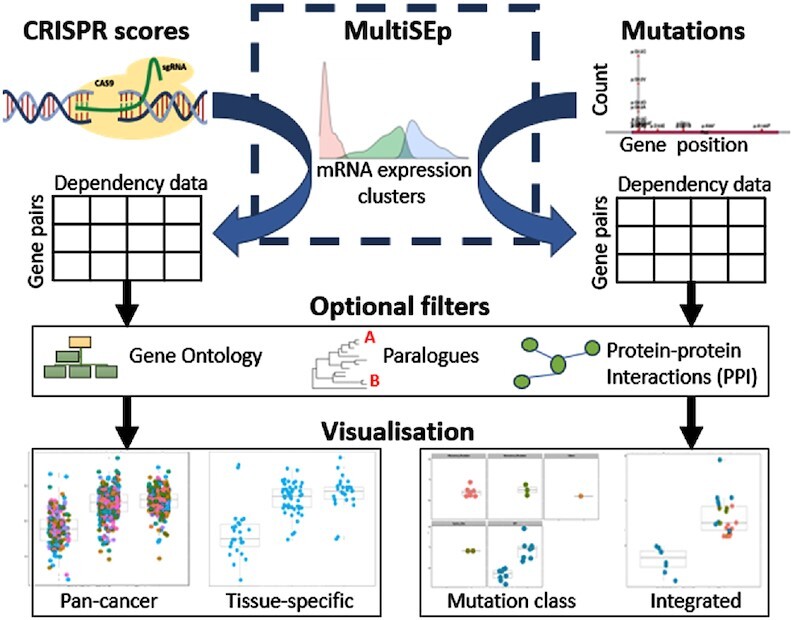
Overview of SynLeGG. CRISPR essentiality scores from CERES (12) and mutations from whole exome sequencing are analysed in separate workflows, partitioned using MultiSEp gene expression clusters. Results are returned as a table where each row describes a gene pair and the columns summarise dependency data, including q-values for the difference between CRISPR or mutation values across the MultiSEp clusters. Application of optional filters enables prioritization of gene pairs with orthogonal evidence of similar function according to common Gene Ontology terms, evolutionary information and protein interactions. Multiple visualizations and download of data are available to facilitate exploration of candidate gene dependency relationships.

SynLeGG incorporates the MultiSEp algorithm for analysis of RNA-Seq data from the Cancer Cell Line Encyclopedia (CCLE) (19). MultiSEp is a refinement of the BiSEp approach (2) which partitions gene expression to discover mutually exclusive loss signatures that are characteristic of synthetic lethality. MultiSEp applies Gaussian mixture modelling (GMM) with Expectation-Maximization to discover gene expression clusters with cardinality determined by Bayesian Information Criterion regularization (21), producing from two up to five clusters of cell lines per gene. Unimodal models are not evaluated because a single cluster would be incompatible with the partitioning required in the downstream analysis. The MultiSEp results for CCLE are available for download within SynLeGG and are summarized in Supplementary Figure S1. The GMM analysis overcomes limitations in the BiSEp approach which splits cell lines into only two groups per gene and only makes comparisons where bimodality is statistically identified (2). MultiSEp predicts pairwise genetic dependencies by partitioning gene effect scores from CRISPR screens or mutational classes using the clusters derived from GMM. For CRISPR dependency relationships, fold-change and two-tailed t-test q-values (22) are calculated between CERES (13) scores for cell lines in neighbouring clusters (Figure 1). Dependencies for mutational data are assessed using a chi-squared test for the enrichment of mutation classes across the MultiSEp clusters (Figure 1).

### Evaluation of MultiSEp performance

We compared the performance of MultiSEp, BiSEp (2) and DAISY (17) on synthetic lethal gene pairs from the SynLethDB database (downloaded November 2020, currently available from http://synlethdb.sist.shanghaitech.edu.cn/) (23). SynLethDB score thresholds were taken to define high confidence (>0.7, *n* = 121) or low confidence (>0.1, *n* = 16916) gold standard synthetic lethal interactions. Resampled gene pairs with no evidence for a synthetic lethal relationship in SynLethDB were taken as the gold standard negatives. The negative pairs might suffer from contamination due to unannotated genetic dependency relationships; however these are rare and so any contamination will be a tiny proportion of the resampled pairs. Predictions from BiSEp and MultiSEp were produced for DepMap RNA-seq, CRISPR data (783 cell lines, version Q3 2020) (1). DAISY predictions were derived from our implementation of the published protocol (17) in R (Supplementary Data File S1) using the DepMap (Q3 2020) copy number and RNA-seq data. We were unable to generate DAISY predictions for genes that did not have copy number losses in at least 2 cell lines across the full panel. Intersecting the gold standard data with predictions from MultiSEp, BiSEp and DAISY produced three separate, balanced, benchmarking datasets (respectively n = 8678, n = 1022, n = 3132). An intersection of predictions from the three methods was unfeasible because of the small number of overlapping gene pairs (*n* = 3, *n* = 22 at SynLethDB thresholds of 0.7, 0.1 respectively). Additionally, overlapping with BiSEp scores would bias results for the other methods due to BiSEp only giving predictions for bimodal data. Therefore, results for each method took every positive gold standard pair from SynLethDB where a prediction was available plus an equal number of negative pairs. Scores for DAISY, MultiSEp were taken as -log *P*-values, fold-change was used for BiSEp. Performance statistics were calculated using the ROCR package (24). For False Discovery Rate estimation, we took the balanced datasets described above, as well as ‘real-world’ datasets with 3.75% SynLethDB gene pairs and 96.25% resampled pairs. These proportions correspond to the frequency of negative genetic dependencies observed in the 5416 genes tested by Costanzo *et al.* (25).

### Implementation

SynLeGG is implemented in a Model-View-Controller architecture (Supplementary Figure S2) respectively consisting of a SQLite database, Shiny user interface and R functions to analyse the DepMap Q3 2020 data (1,13,19). Each user session is deployed in a separate Docker container managed by Shinyproxy behind an NGINX reverse proxy, enabling strong performance at scale. The database consists of 8 tables (Supplementary Figure S3), and is designed for read speed to enhance the user experience.

### USAGE

Figure 1 gives an overview of the steps involved in using SynLeGG. Tabs for analysis of CRISPR or mutation data are accessed from links in the navigation bar that appears on every page, which also links to extensive help documentation. Context-sensitive help is available from tooltips and as pop-ups that provide focussed extracts from the user guide, accessed by clicking green information icons at the top right of each subsection of the website. The documentation includes a tutorial and quick start guide. Demonstration mode is activated from a checkbox within the ‘CRISPR’ or ‘Mutation’ tabs, and walks users through the key features of SynLeGG. After navigating to www.overton-lab.uk/synlegg, the first step is to click the ‘Launch’ button which loads a unique Docker container for the SynLeGG session; then select either ‘CRISPR’ or ‘Mutation’ in the navigation bar.

### Analysis of CRISPR scores and gene expression to propose gene dependency relationships

**Figure 2.**
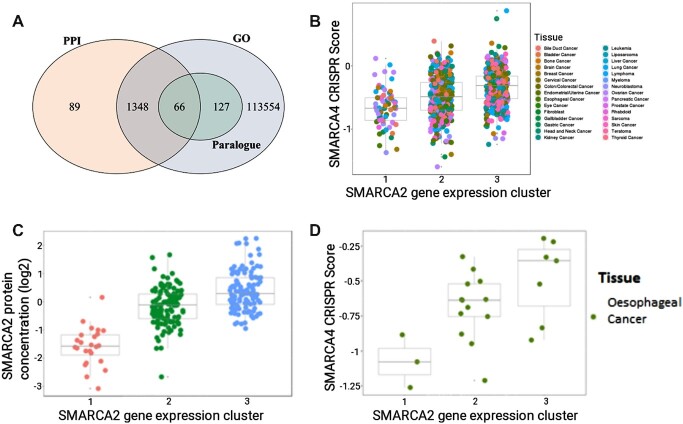
Dependencies between CRISPR essentiality scores and MultiSEp clusters. (**A**) Venn diagram showing 115,184 gene pairs that pass the SynLeGG inclusion thresholds (*P*< 0.1, log_2_ fold-change > 0.1) and have overlapping Gene Ontology (GO) annotations, protein–protein interactions (PPIs) or are Ensembl human paralogues. As might be expected, all 193 paralogue pairs have a common GO term. (**B**) SMARCA4 CRISPR scores are visualized within MultiSEp clusters for SMARCA2 across 783 cell lines, coloured by thirty tissue groupings (see key). Essential and non-essential genes have median CRISPR scores of -1 and 0, respectively. As expected, SMARCA2 gene expression correlates with SMARCA4 CRISPR score; cell viability or growth is most damaged by loss of SMARCA4 in cell lines within low SMARCA2 expression clusters. (**C**) SynLeGG provides visualization of mass spectrometry proteomics data, where available. The figure shows SMARCA2 protein concentrations for the MultSEp gene expression clusters. The distribution of protein concentrations within each cluster follows the same trend as the mRNA measurements in matched cell lines, for example cluster 1 left) has low expression, providing evidence for chemical inhibition of SMARCA2 as a viable therapeutic strategy in cancers with low SMARCA4 activity. (**D**) The synthetic lethal relationship between SMARCA2 and SMARCA4 is shown for oesophageal cancer cell lines, accessed using the ‘Tissue Type’ mode in SynLeGG.

The ‘CRISPR’ tab, located in the navigation bar, provides a results table with integrated MultiSEp analysis of gene expression and CRISPR scores for investigation of candidate ‘Achilles Heel’ relationships. The default ‘All Tissue’ mode offers pan-cancer analysis across 783 cell lines and the ‘Tissue Type’ section provides analysis within a selected tissue. Checkboxes allow optional filtering of results according to evidence of functional similarity from overlapping Gene Ontology (GO) annotations (26,27), BioGRID physical protein-protein interactions (PPIs) (28) and Ensembl human paralogues (29,30). A total of 169 172 gene pairs are available within the CRISPR tab, passing the thresholds of log_2_ fold-change >0.1, *P*-value <0.1. Of these, 115 095 have at least one shared GO term, 1503 have at least one PPI and 193 are paralogous (Figure 2A). Selecting a gene pair in the ‘Results’ table displays information in the ‘Details’ table and visualizes results in the ‘Plot’ section. For example, searching for SMARCA2 in the ‘mRNA_gene’ column and for SMARCA4 in the ‘crispr_gene’ column returns an established synthetic lethal interaction within the SWI/SNF complex (8,9). SynLeGG affirms that knockout of SMARCA4 by CRISPR is more damaging for cell lines with low SMARCA2 gene expression (Figure 2B). Mass spectrometry proteomics (20) is present for a subset of the cell lines and genes analysed by MultiSEp; where available, SynLeGG enables exploration of the concordance between protein concentrations and mRNA expression to help inform candidate drug target prioritization (Figure 2C). Tissue-specific analysis shows that the dependency between SMARCA4 and SMARCA2 is particularly robust in oesophageal cancer cell lines (Figure 2D), consistent with previous findings (31).

### Interrogating mutations and gene expression data to reveal candidate gene dependencies

**Figure 3.**
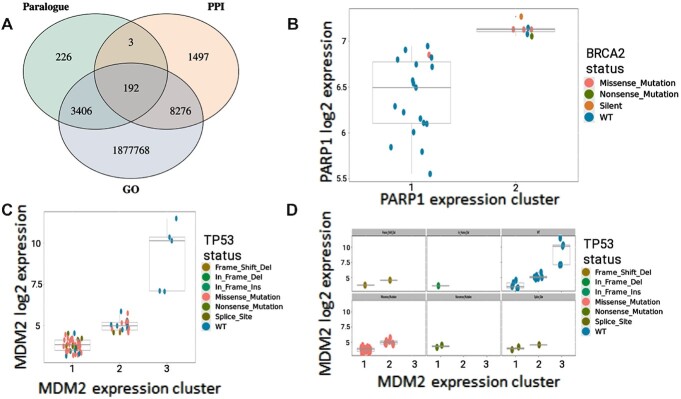
SynLeGG enables exploration of relationships between gene expression clusters and mutational status. (**A**) Venn diagram showing 1 891 368 gene pairs with candidate dependencies between mutations and MultiSEp clusters (*P*< 0.05, ≥5 mutations), which also have overlapping Gene Ontology (GO) annotations, protein-protein interactions (PPIs) or are Ensembl human paralogues. (B–D) visualize established dependency relationships between gene expression and mutational status. Gene expression values are given on the y-axis and MultiSEp clusters are indicated on the x-axis. Each cell line is coloured by mutational status according to the key. (**B**) shows an established synthetic lethal relationship between BRCA2 and PARP1 in oesophageal cancer cell lines. As expected, cell lines with low PARP1 expression are BRCA2 wild-type (blue), while the majority of cell lines with high PARP1 expression have BRCA2 mutations. (**C**) and (**D**) identify an ‘induced dependency’ relationship between TP53 mutations and MDM2 expression in brain cancer cell lines. MDM2 is a negative regulator of TP53 and all of the cell lines with high MDM2 expression are TP53 wild-type (blue); conversely the greatest proportion of TP53 mutations are found in the lowest MDM2 expression cluster. These data underline the attractiveness of MDM2 inhibitors in TP53 wild-type cancers. Separate plots for different mutation types are shown in (D); six plots appear in the Figure, however up to nine may be shown in SynLeGG.

Analysis of integrated mutations and gene expression data is available within SynLeGG from the ‘Mutation’ tab in the navigation bar. Results are obtained by typing one or more gene symbols into the ‘Mutation Gene(s)’ box at the top left; if the entered text is not recognized, a dictionary lookup of synonyms may be activated with the ‘Check Symbol(s)’ button. SynLeGG includes 3 692 429 candidate dependency relationships with *P* <0.05 and ≥5 mutations in the ‘Mutation Gene’ across all tissue types, of which 1 889 642 have a common GO Term, 9968 have PPIs and 3827 are paralogues (Figure 3A). The ‘Mutation Results’ table shows candidate gene expression dependency relationships predicted by MultiSEp for the ‘Mutation Genes’ and resolved by tissue; selecting a gene pair visualizes results in the ‘Plot’ section and populates the ‘Mutation Details’ table with information about shared GO terms, PPIs and paralogy. The results can be filtered on all columns and sorted by tissue type, *q*-value or number of mutations. The well known synthetic lethal relationship between BRCA2 and PARP1 (6,7) is visualized for oesophageal cancer cell lines in Figure 3B, which shows that BRCA2 mutations are absent when PARP1 has low expression. We note that SynLeGG analyses somatic mutation calls, however synthetic lethality may involve germline changes; indeed inherited BRCA mutations occur at an appreciable frequency (5). Therefore, the exclusion of germline mutations is a current limitation for the exploration of genetic dependencies with SynLeGG and could explain why cell lines annotated as wild-type may appear in expression clusters enriched for deleterious somatic mutations; the ‘wild-type’ allele might represent a deleterious germline mutation (1). In addition to synthetic lethality, other relationships that may represent Achilles heels can be identified using SynLeGG, including candidate induced dependency. For example, TP53 mutations are depleted in brain cancer cell lines with high MDM2 expression (Figure 3C, D). MDM2 is a negative regulator of TP53 and so elevated MDM2 relieves the selection pressure for inactivating mutations in TP53 (32). Therefore, inhibitors against MDM2 may be effective in cancers with high MDM2 expression and wild-type TP53 (32).

### Benchmarking MultiSEp

MultiSEp, BiSEp and DAISY respectively had Area under the Receiver Operator characteristic Curve (AROC) values of 0.71, 0.59, 0.39 for SynLethDB gene pairs scoring >0.7 (28); and AROC of 0.57, 0.53, 0.5 respectively at SynLethDB threshold >0.1 (Supplementary Figure S4, Supplementary Table S1). MultiSEp had significantly higher AROC values than DAISY at both SynLethDB thresholds examined (>0.7, *P* < 0.019; >0.1, *P* < 5.4 × 10^−9^) and significantly (33) outperformed BiSEp at the lower threshold (*P* < 0.036). Few predictions were available from DAISY (*n* = 10) and BiSEp (*n* = 11) at the high-confidence SynLethDB threshold, making statistical comparisons more difficult. MultiSEp coverage is respectively 5.8-fold, 6.4-fold higher than BiSEP and DAISY at SynLethDB threshold >0.7 and 8.5-fold, 2.8-fold higher for SynLethDB gene pairs scoring >0.1. Therefore, MultiSEp provides better performance over a much larger number of candidate gene pairs. We also assessed the effect of optional filtering upon MultiSEp performance (Supplementary Figure S4). Filtering by common GO terms did not significantly affect MultiSEp performance, which may be expected due to the inclusion of high level terms; however, GO information within SynLeGG provides useful context. Filtering by PPIs significantly increased performance relative to unfiltered MultiSEp analysis at the lower SynLethDB threshold value, although with 9.7-fold lower coverage (449 pairs, AROC 0.66, *P* < 2.3 × 10^−6^). Taking only paralogue pairs dramatically reduced coverage, by 98.7-fold, with a trend towards better performance relative to no filtering at the lower SynLethDB threshold (44 pairs, AROC 0.68, *P* < 0.0547). We also assessed false discovery rate (FDR) using SynLethDB, on both the balanced datasets and taking a ‘real-world’ proportion of genetic dependencies (Supplementary Figure S5). MultiSEp performed best and, reassuringly, stricter thresholds result in better FDR values. A total of 24 CRISPR predictions from SynLeGG overlapped with a recently published screen (34), where 18/24 (75%) had Bonferroni-corrected *T*-test *P*-value <10^−5^; corresponding to FDR = 0.25 (Supplementary Table S2).

## CONCLUDING REMARKS

Large CRISPR screens, exome sequencing and RNA-seq datasets provide unprecedented opportunities for cancer drug target prioritization and to discover new gene functions (1,13,19). We integrate these resources for nomination of candidate Achilles’ heel relationships, where the status of one gene exposes a cell's vulnerability to the perturbation of a second gene. Our approach is validated at scale on data from SynLethDB (23), compared against BiSEp (2) and DAISY (17), and exemplified with gold-standard published synthetic lethal relationships. A recent pairwise screen focussed on paralogues validated 18 of 24 (75%) overlapping pairs (34), representing significant enrichment of validated pairs in the SynLeGG predictions (two-tailed FET *P* < 2.8 × 10^−10^). Pairwise genetic dependencies are highly sensitive to biological context and are frequently modified by a third gene (35); therefore the genetic dependency relationships that did not validate in the Thompson *et al.* study but are predicted by SynLeGG might manifest in cell lines other than the three examined in (34). SynLeGG provides access to our integrated approach for the wider scientific community, enabling analysis and visualization of genetic dependency relationships across 783 cell lines and 30 tissues. Key features are the partitioning of essentiality scores (13) or mutational classes within MultiSEp gene expression clusters, and the investigation of tissue-specific gene dependencies. Relationships identified with the RNA-based clusters may be explored in available mass spectrometry proteomics data, a useful component of drug target prioritization. Results are integrated with complementary information to inform gene functional similarities from the Gene Ontology (26,27), protein-protein interactions (28) and evolutionary information (29,30). SynLeGG has been successfully tested on multiple web browsers (Firefox, Chrome, Edge, Safari) and major operating systems (Linux, MacOS, Windows). We very much appreciate feedback on any issues relating to SynLeGG, ideally sent using the form accessible from the ‘Contact’ tab in www.overton-lab.uk/synlegg and we welcome requests for new functionality.

## DATA AVAILABILITY

SynLeGG is available at www.overton-lab.uk/synlegg. It is a free web-based service open to all users and there is no login requirement. Our implementation of DAISY (17) is available in Supplementary Data S1.

## Supplementary Material

gkab338_Supplemental_Files
