# Supplementary material for: SynLeGG: analysis and visualization of multiomics data for discovery of cancer ‘Achilles Heels’ and gene function relationships
Source: Nucleic Acids Res. 2021 May 17;49(W1):W613–8. doi: 10.1093/nar/gkab338 (PMC8265155; doi:10.1093/nar/gkab338)
Supplement: gkab338_Supplemental_Files [file gkab338_supplemental_files.zip › Wappett_etal_Supplementary_Information.pdf]

Supplementary Information for "SynLeGG: Analysis and Visualisation of Multiomics Data for Discovery of Cancer 'Achilles Heels' and Gene Function Relationships"

Mark Wappett, Adam Harris, Alexander L. R. Lubbock, Ian Lobb, Simon McDade & Ian M. Overton

Contents Page

Figure S1: Overview of MultiSEp Clustering Results 2

Figure S2: Outline SynLeGG application architecture 3

Figure S3: Simplified database schema 3

Figure S4: MultiSEp, BiSEp and DAISY Performance on SynLethDB Benchmark Data 4

Figure S5: False Discovery Rate Profiles for MultiSEp, BiSEp and DAISY 5

Table S1: Summary of SynLethDB benchmarking results 6

Table S2: Independent validation of SynLeGG predictions for paralogue pairs 7

Supplementary Data S1 contains R code and is provided as a separate file.

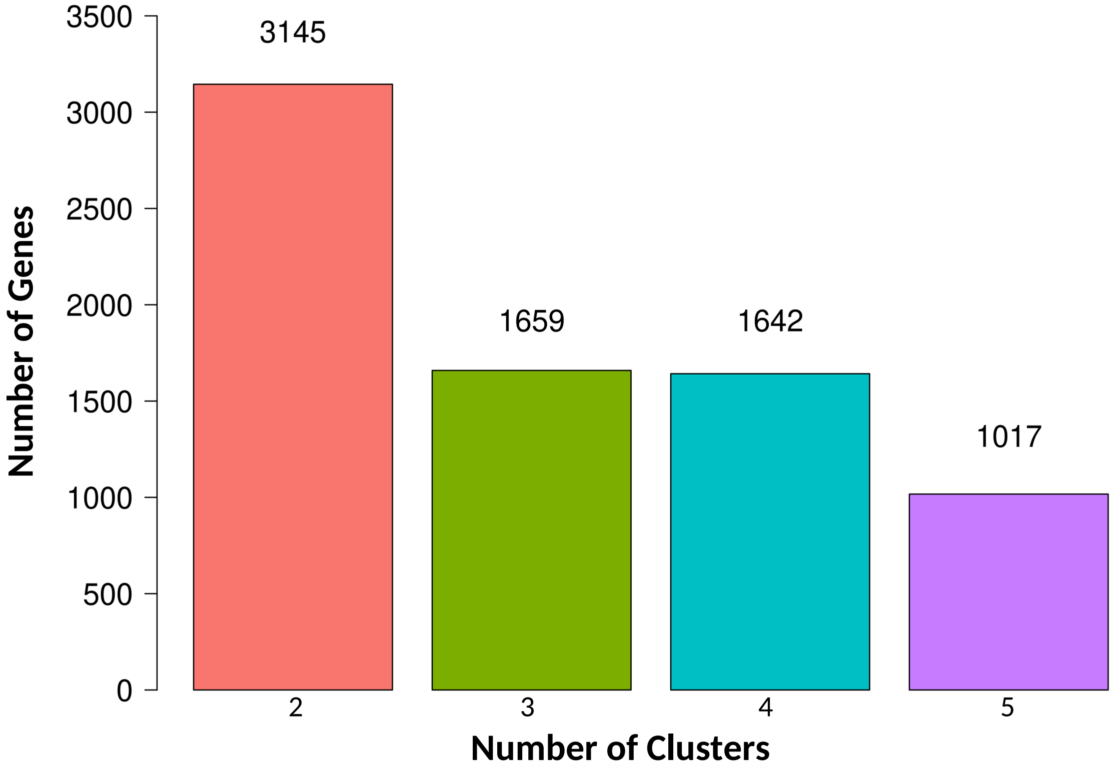

Supplementary Figure S1: Overview of MultiSEp Clustering Results. The gene expression clusters identified by MultiSEp in SynLeGG are shown on the X-axis, between two and five clusters may be determined across the 783 cell lines analysed. The number of genes in each cluster is given on the Y-axis and shown above each bar. The highest number of genes fall into two gene expression clusters (n=3145), however the majority of genes have more than two clusters (n=4318). Only genes with CR-ISPR data in SynLeGG are shown.

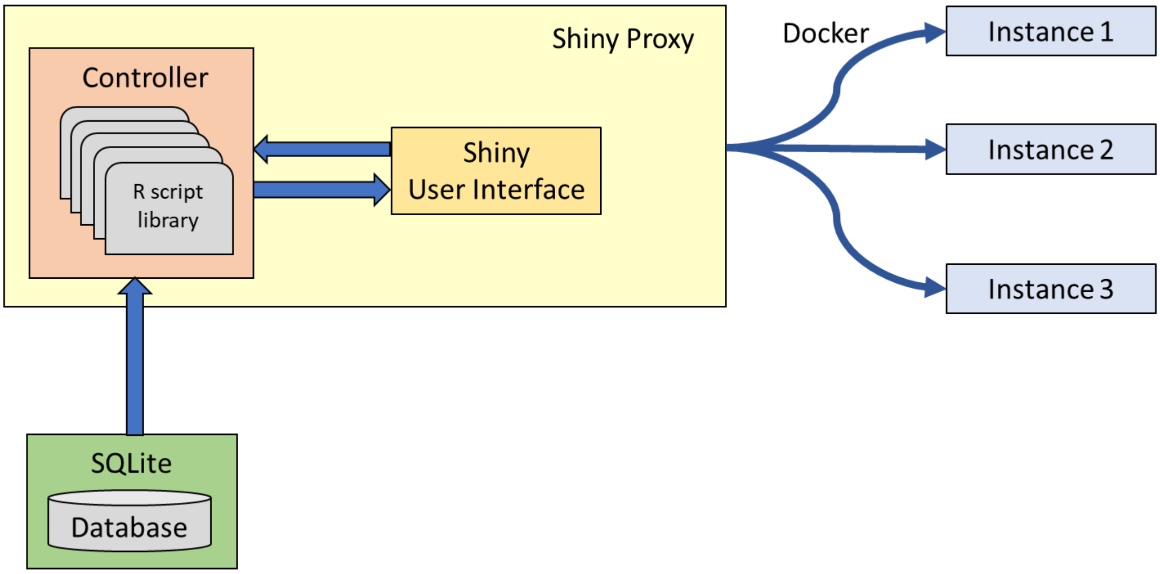

Supplementary Figure S2. Outline SynLeGG application architecture. SynLeGG utilises Shiny Proxy, instantiating a Docker container for each user session. The controller interacts with the Shiny User interface and reads from the SQLite database.

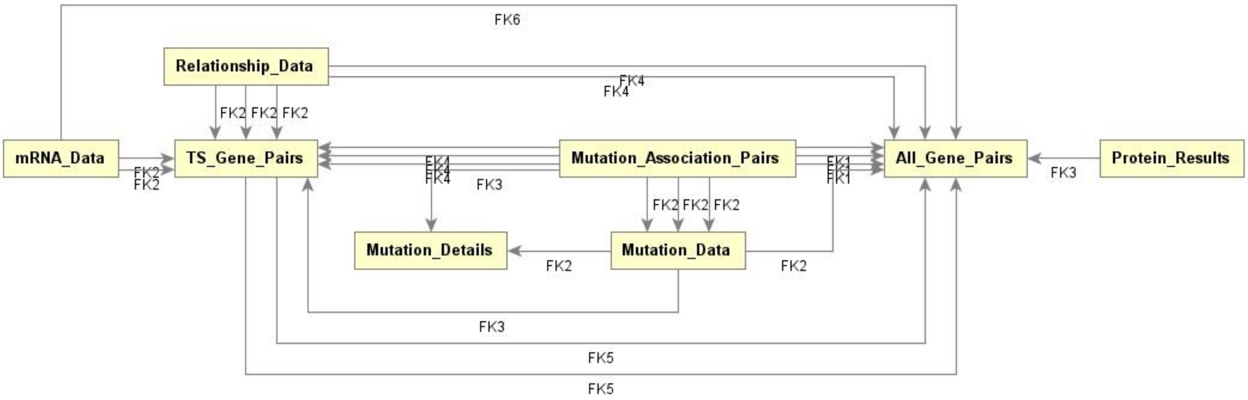

Supplementary Figure S3. Simplified Database Schema. The eight database tables are shown, arrows represent foreign keys.

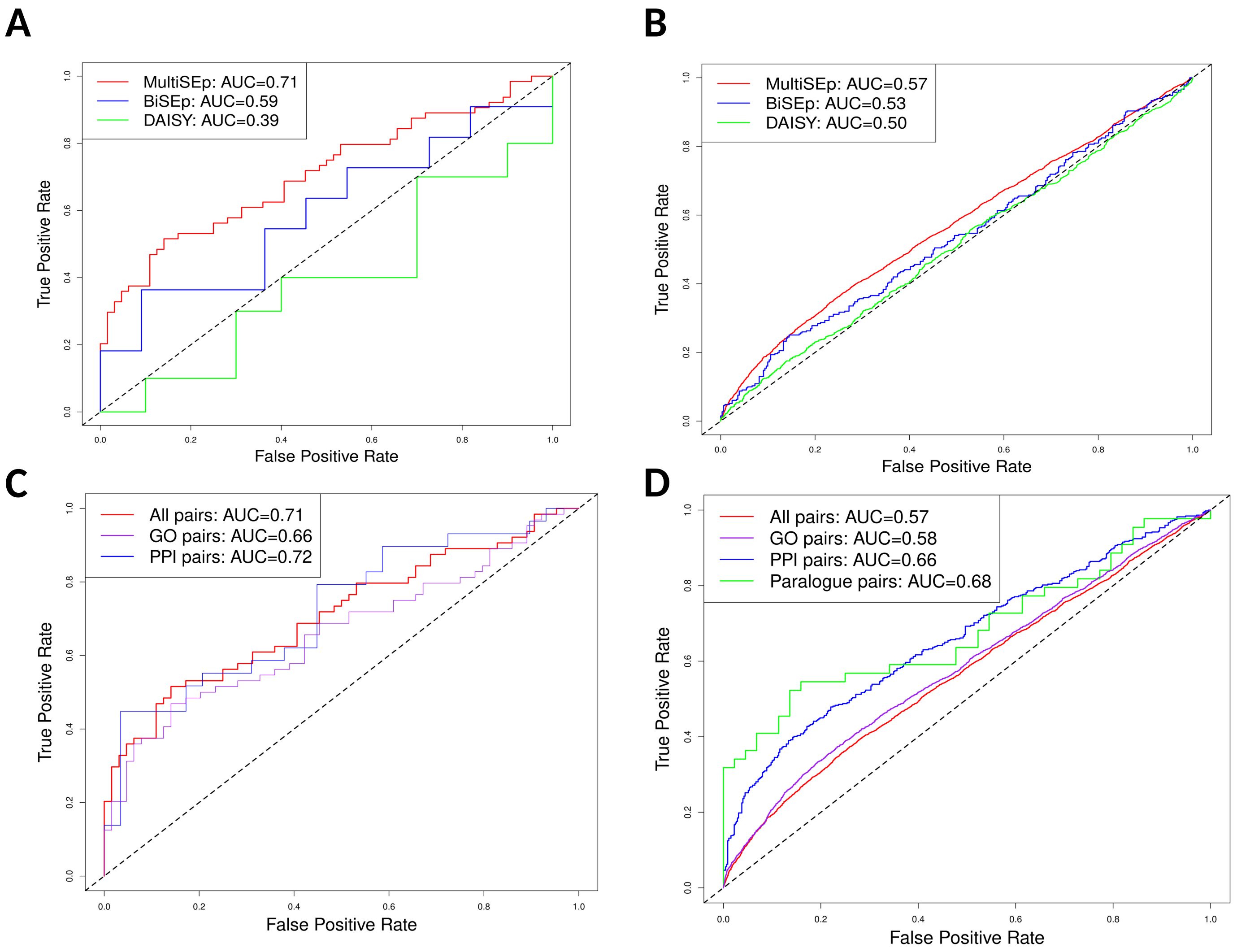

Supplementary Figure S4. MultiSEp, BiSEp and DAISY Performance on SynLethDB Benchmark Data. Receiver Operator Characteristic (ROC) curves are shown for MultiSEp to BiSEp and DAISY predictions on gene pairs taken from SynLethDB at thresholds of 0.7 (A) and 0.1 (B). MultiSEp performs best overall. The SynLethDB threshold of 0.1 includes predictions from DAISY, which may artificially inflate DAISY performance in (B). Additionally, ROC curves are shown for either unfiltered MultiSEp predictions (‘All pairs’), filtering using common Gene Ontology (GO) terms, by BioGrid Protein Interactions (PPI) or with Ensembl human paralogues at SynLethDB threshold of 0.7 (C) and 0.1 (D). Results are not shown for paralogue filtering with a threshold of 0.7 because there was only one paralogue pair that had a MultiSEp prediction and SynLethDB score >0.7. The PPI and paralogue filtering appears to increase performance, although reduces coverage. The dashed diagonal line in each plot shows expected baseline performance for a random predictor, corresponding to an AUC value of 0.5. Please see the main manuscript and Supplementary Table S1 for further details.

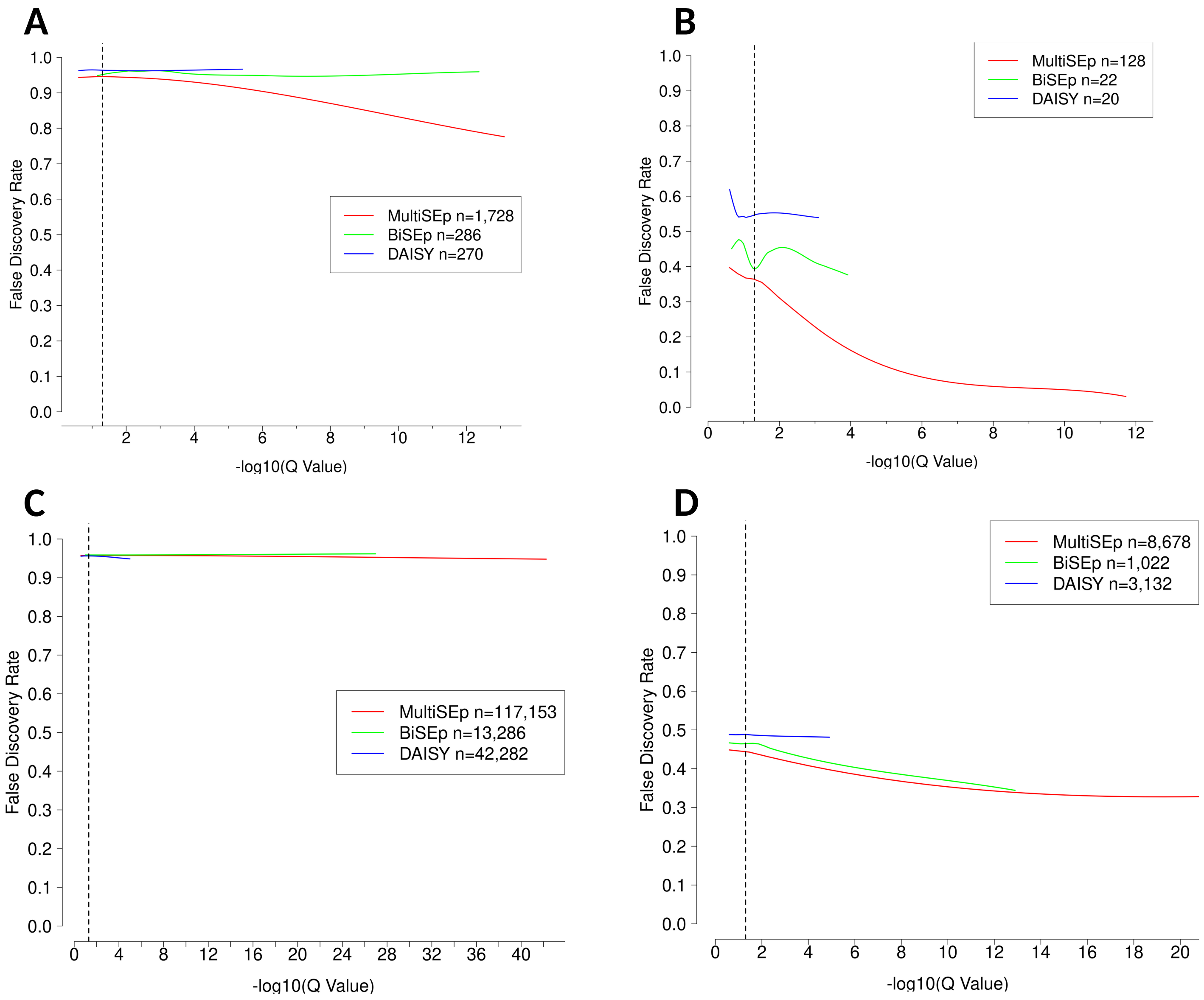

Supplementary Figure S5: False Discovery Rate Profiles for MultiSEp, BiSEp and DAISY on SynLethDB. False Discovery Rate is shown against the q-value threshold; SynLethDB gene pairs form the positive class and the negative class is composed from resampled gene pairs. The vertical dashed line shows q=0.05. The total number of positive and negative pairs analysed by each algorithm is given in the Figure key. (A) and (C) respectively show results for SynLethDB thresholds of 0.7 and 0.1 where the benchmark data has a 'real-world' proportion of positives (3.75%). This 'real-world' proportion was determined with reference to Costanzo *et al.* ([25] in the main manuscript). (B) and (D) respectively show results for SynLethDB thresholds of 0.7 and 0.1 where the benchmark data has equally balanced positive and negative gene pairs (50:50). MultiSEp has lowest FDR overall and, reassuringly, the best FDR is found at the most significant q-values. The fluctuations in the relationship between FDR and -log(q) shown in (B) for DAISY and BiSEp likely reflects the small dataset size (20 pairs total for DAISY, 22 pairs for BiSEp). We note that the lower SynLethDB theshold of 0.1 in (C) and (D) includes DAISY predictions as positive gene pairs, which could artificially enhance DAISY performance. Indeed, the 0.7 threshold represents 'high-confidence' gene pairs and so may provide the most reliable performance estimates. Filtering gene pairs with known protein-protein interactions or paralogues may produce a performance improvement, as observed in Supplementary Figure S4.

Algorithm	SynLethDB threshold	Filter	# SynLethDB gene pairs	AROC	SynLethDB pairs with predictions
BiSEp	0.7	-	121	0.59	11
BiSEp	0.1	-	16916	0.53	511
DAISY	0.7	-	121	0.39	10
DAISY	0.1	-	16916	0.5	1566
MultiSEp	0.7	-	121	0.71	64
MultiSEp	0.1	-	16916	0.57	4339
MultiSEp	0.7	PPI	75	0.72	29
MultiSEp	0.1	PPI	1285	0.66	449
MultiSEp	0.7	Paralogue	3	NA	1
MultiSEp	0.1	Paralogue	53	0.68	44
MultiSEp	0.7	GO	116	0.66	64
MultiSEp	0.1	GO	11705	0.58	3607

Supplementary Table S1. Summary of SynLethDB benchmarking results. MultiSEp was compared against BiSEp and DAISY using the SynLethDB database (V2). A high confidence score threshold was used on SynLethDB (0.7) otherwise all gene pairs were used (0.1). The effect of filtering for PPI, Paralogue and GO gene pairs on performance was also assessed for MultiSEp. The number of gene pairs with predictions (final column) represents the subset of pairs in the '#SynLethDB gene pairs' column that have predictions from the software shown in the 'Algorithm' column. For example, BiSEp only makes predictions where a bimodal gene expression distribution can be identified statistically - limiting the total number of predictions available. The unfiltered MultiSEp predictions have the highest number of predictions and the best AROC value at both SynLethDB threshold values examined ('high-confidence', 0.7; 'low-confidence', 0.1). Filtering reduces coverage but the PPI and Paralogue filters increase performance at the 'low-confidence' threshold.

mRNA Gene	CRISPR Gene	MultiSEp q-value	Validated: (T-test)*
ATP6V1B1	ATP6V1B2	1.49×10^{-04}	Yes
CDS1	CDS2	1.06×10^{-20}	Yes
DNAJC6	GAK	1.47×10^{-02}	Yes
EAF2	EAF1	3.23×10^{-35}	Yes
ELL2	ELL	1.36×10^{-08}	Yes
FAM50B	FAM50A	6.15×10^{-31}	Yes
RPP25	RPP25L	2.97×10^{-42}	Yes
RRAGD	RRAGC	3.66×10^{-07}	Yes
SLC25A37	SLC25A28	3.49×10^{-12}	Yes
SMARCA2	SMARCA4	1.78×10^{-07}	Yes
TTC7B	TTC7A	4.71×10^{-21}	Yes
UAP1L1	UAP1	3.37×10^{-20}	Yes
ATP6V1C2	ATP6V1C1	9.01×10^{-05}	Yes
CAP2	CAP1	2.82×10^{-11}	Yes
CTBP2	CTBP1	1.58×10^{-05}	Yes
ENO2	ENO1	3.88×10^{-10}	Yes
PSMB9	PSMB6	2.07×10^{-15}	Yes
STRIP2	STRIP1	1.57×10^{-10}	Yes
CAB39L	CAB39	3.63×10^{-04}	No
CAND2	CAND1	3.54×10^{-02}	No
CTPS2	CTPS1	2.58×10^{-02}	No
CYB5A	CYB5B	2.88×10^{-10}	No
EFR3B	EFR3A	7.50×10^{-15}	No
NMT2	NMT1	2.53×10^{-37}	No

*Bonferroni-corrected T-test $p<10^{-5}$ in at least one time point and at least one cell line from Thompson *et al.* 2021 (reference [34] in the main manuscript).

Supplementary Table S2: Independent validation of SynLeGG predictions for paralogue pairs. SynLeGG provides predictions for 193 paralogous gene pairs at generous MultiSEp thresholds ($p<0.1$, log2 FC>0.1), of which twenty-four were assessed in the Thomson *et al.* combinatorial CRISPR screen (reference [34] in the main manuscript). This table identifies eighteen pairs predicted by SynLeGG that had highly significant Bonferroni-corrected T-test p-values in the Thompson *et al.* study and six gene pairs that were not significant. Therefore, 18/24 (75%) of the overlapping SynLeGG predictions

were validated, corresponding to a false discovery rate of 0.25. The SynLeGG results derive from analysis of 783 cell lines, however the Thompson *et al.* CRISPR screen was performed using three cell lines. Therefore, is possible that the genetic dependency relationships predicted by SynLeGG manifest in cell lines that were not tested by Thompson *et al.*. Indeed, pairwise genetic dependencies are highly sensitive to biological context and are frequently modified by a third gene (please see reference [35] in the main manuscript).
